# One-Point Calibration of Low-Cost Sensors for Particulate Air Matter (PM) Concentration Measurement

**DOI:** 10.3390/s25030692

**Published:** 2025-01-24

**Authors:** Luigi Russi, Paolo Guidorzi, Giovanni Semprini, Arianna Trentini, Beatrice Pulvirenti

**Affiliations:** 1 Department of Industrial Engineering, Alma Mater Studiorum Università di Bologna, Viale Risorgimento 2, 40136 Bologna, Italy; luigi.russi@studio.unibo.it (L.R.); paolo.guidorzi@unibo.it (P.G.); giovanni.semprini@unibo.it (G.S.); 2 Technical Direction, ARPAE Emilia-Romagna, L.go Caduti del Lavoro, 6, 40122 Bologna, Italy; atrentini@arpae.it

**Keywords:** air quality, low-cost sensors, sensor validation, pollutant concentration measurement

## Abstract

The use of low-cost sensors has dramatically increased in recent years in all engineering sectors. In the buildings and automotive field, low-cost sensors open very interesting perspectives, because they allow one to monitor temperature and humidity distributions together with air quality in a widespread and punctual way and allow for the control of all energy parameters. The main issue remains the validation of the measurements. In this work, we propose an innovative approach to verify the measurements given by some low-cost systems built ad hoc for automotive applications. Two independent low-cost measurement systems were set to measure Particulate Air Matter (PM) concentration, TVOC concentration, CO_2_ concentration, formaldehyde concentration, air temperature, relative humidity, pressure, air flow velocity, and GPS position. These systems were calibrated for PM concentration measurement by comparison with standard and certified sensors used by the regional authority of the Emilia-Romagna region (ARPAE, Italy) for characterizing air quality. The duration of the analysis, three days, is not representative of the diverse environmental conditions that occur across different seasons. However, the innovation of this approach lies in both the in-field comparison of low-cost and high-quality sensors and the use of proper conversion approaches for mass concentration measurements. A quantitative analysis of the sensors’ performance is given, with a focus on the effects of time granularity, relative humidity, mass conversion from particle counts, and size detection response. The results show that the low-cost sensors’ measurements of air temperature, relative humidity, and particle number concentration are in good agreement with high-quality sensors’ measurements, with a strong impact of relative humidity on performance indicators. Overall, good quality and consistency of the data among the sensors were achieved.

## 1. Introduction

Interest in air quality monitoring is gaining more and more attention from public authorities, companies, and citizens for both outdoor and indoor environments [1,2]. Climate change, pollution, and COVID-19 issues are well-known air quality-related topics that are very important in buildings, but the automotive sector is also pushing the development of novel air quality standards. Several studies have underlined the relevance of particle counters in the determination of air pollution that adversely affects health, thus suggesting that both particle number and mass concentrations should be measured [3]. The diffusion of low-cost sensors is considered a promising technology for improving the spatial and temporal resolution of these measurements. Okorn et al. [4] present an extended overview of outdoor low-cost gas-phase air quality sensor network deployments, showing disparities on global and regional scales. They found that the highest-quality sensor data are obtained by utilizing an outdoor collocation with a reference instrument and subsequent algorithm development to ensure a proper fit. They also found that the larger the network is and the more area is covered, the less likely it is that every node has been properly calibrated. However, it must be noted that the accuracy of the data obtained from these devices is questionable compared to reference measurement techniques and is still a matter of research [5]. The United States Environmental Protection Agency held an international two-day workshop in June 2018 to deliberate possible performance targets for non-regulatory fine particulate matter (PM2.5) and ozone (O3) air sensors [6]. The need for a workshop arose from the lack of any market-wide manufacturer requirement for documented sensor performance evaluations, the lack of any independent third party or government-based sensor performance certification program, and uncertainty among all users as to the general usability of air sensor data. A key message was that a number of possible future actions would be beneficial to all stakeholders regarding sensor technologies. These included documentation of best practices, sharing quality assurance results along with sensor data, and the development of a common performance target lexicon, performance targets, and test protocols. The WMO Report No. 121 [7] provides a comprehensive update on the use and operation of low-cost sensing devices for assessing atmospheric chemical composition. In the report, the differences between assessing sensors in the laboratory and in the field are outlined. In the report, important factors like the duration of the calibration and the influence of temperature, relative humidity, and cross-sensitive gas species are considered. The most recent works in the literature show that efforts in the development [8] and performance assessment [9] of low-cost sensors have been made at both the sensor and system levels, emphasizing the importance of calibration for reliable output. A comparison of the sensor data generated by different microsensor systems installed side-by-side with reference analyzers has been shown by Borrego et al. [10,11]. The experiments contributed to the assessment of the performance and the accuracy of microsensor systems in a real-world context, and supported their calibration and further development. The results confirmed that the microsensor platforms, supported by post-processing and data modeling tools, have considerable potential in new strategies for air quality control. Regarding the sensors, Belosi et al. [12] evaluated the performance of four optical particle counters (OPCs) with a standardized particle generator; they found good results for the total particle number concentration, while the aerosol size distribution and average particle density must be improved as they are closely related to the particle mass concentration [12]. Conversion from particle-to-mass concentration represents a crucial task if good performance of OPCs is desired. A study by Franken et al. compared different conversion methods with a focus on PM2.5 mass concentrations [13]. They found that while good correlations (Pearson) are possible with the developed method, other methods resulted in an underestimation of particle mass concentration if compared with gravimetric data. However, many manufacturers only perform calibration in environmental chambers with controlled process parameters. The latter is a necessary but not sufficient procedure, thus indicating field calibration as a mandatory task, particularly if the acquisition system needs to be relocated. Recent studies have investigated the performance of different calibration approaches, ranging from simple linear regression to machine learning (ML) techniques [14]. The selection of the best method is strongly case-specific and must be performed considering experiment setup, sensor selection, site location, and measurement duration. An example of field calibration is given by Dinoi et al. [15]. The work reports a comparison between three OPCs against an urban background reference station, showing that the effect of relative humidity (RH) is considerable and should be compensated for, especially for mass concentration evaluation. In contrast, Zou et al. investigated the relationship between environmental variables such as air temperature and RH in eight low-cost particle sensor outputs [16], but using a controlled chamber and common particle sources. On the one hand, they found no significant effect of temperature; on the other hand, RH has an impact on the magnitude of particle readings but still may be compensated with a simple RH-based calibration as the output correlates well with reference instrumentation. If a vehicle cabin IAQ LCSoS is considered mobile, it is therefore a continuously relocated system; the robustness of its field calibration method becomes of paramount importance. Existing assessment techniques rely on relevant factors’ probability distribution changes that allow for the performance prediction of field calibration models, but co-located reference measurements involving several months are still required [17]. Although low-cost alternatives are gaining popularity, their reliability is still an issue, due to their sensitivity to environmental conditions, inherent instability, and manufacturing imperfections. Koziel et al. [18] applied artificial intelligence methods to field calibration for low-cost PM sensors, obtaining a custom-designed portable PM monitoring platform and reference data acquired from public measurement stations. Even if these techniques are promising, there is still a need for studies on field calibration techniques. The main aim of this work is to build and perform a fast field calibration of an Arduino-based low-cost SoS at the system scale. The effects of environmental variables on particle-related output variables are also discussed. Secondly, our objective is to assess the performance, at the sensor scale, of SPS30, a quite new and powerful device that has still poor research support. The third objective is to demonstrate the metrological capabilities of an LCSoS in the automotive field, thus enabling its operation in transient, non-uniform, and moving environments such as vehicle cabins [19]. The study presents a short-period calibration technique that demonstrates excellent agreement with reference measurements. The results indicate strong performance concerning air temperature, relative humidity, and particle number concentration, achieving a determination coefficient approaching 0.92 for the reduced data set.

## 2. Materials and Methods

In this section, to describe the complex in-field methodology deployed for the validation of low-cost sensors, the following subsections are presented. First, two low-cost sensor arrangements built for the characterization of air quality are introduced. Then, the site with the reference-calibrated instrumentation used by the regional authority ARPAE is described. The details of the measurement campaign with the low-cost sensor arrangement at the reference station are explained. Finally, the methodologies used to acquire and manipulate the data for comparison, such as time granularity and mass conversion, are discussed.

### 2.1. Arduino-Based Sensor Systems

Two independent measurement systems (IS and ES) based on Arduino Mega 2560, (manufactured in Italy, Turin, by Arduino srl), were built for the measurement of environmental parameters [20,21]. The systems have a onboard Real-Time Clock (RTC), a data logger on flash memory, a fan, and a TFT display. The RTCs of the two systems are constantly synchronized thanks to time data received from the GPS module. Both systems can measure the concentration of particles in air matter (PM) (Sensirion SPS30 sensor, manufactured in Zurich, Switzerland, by Sensirion AG), the concentration of TVOC in the air (Sensirion SGP30 sensor), the concentration of air CO_2_ (Winsen MH-Z19B nondispersive infrared sensor, manufactured in Zhengzhou, China, by Zhengzhou Winsen Electronics Technology Co., Ltd.), the formaldehyde concentration (Winsen ZE08 sensor, manufactured in manufactured in Zhengzhou, China, by Zhengzhou Winsen Electronics Technology Co., Ltd.), the temperature, relative humidity and pressure of the air (Bosch Sensortec BME280 sensor, manufactured in Reutlingen, Germany, by Bosch Sensortec GmbH), air flow velocity (hot wire analog sensor), and GPS position. The systems are equipped with a fan that transports air inside the device enclosure, where CO_2_ and formaldehyde sensors are mounted, while the SPS30 sensor is equipped with its built-in fan. Both systems independently sampled data in 10 s intervals. All digital sensors used in the measurement device include a microcontroller that implements optimization and self-calibration algorithms. Among all the quantities measured by the low-cost SoS, only some sensors have been validated, i.e., those that overlap with the reference instrumentation. These are shown in Table 1, together with the available daily values from the ARPA reference station. The BME280 sensor was used for temperature and humidity measurements, while the SPS30 sensor was used for PM concentration measurements in the LCSoS. In Table 1, IS and ES are the two independent LCSoSs, while AS refers to the equivalent equipment measurement system of the ARPAE considered for validation. In Table 1, MSR means the Main Site Reference data, i.e., the daily measurements obtained by the ARPAE reference equipment [22].

AS provided measurements that are comparable in accuracy and reliability to those of the reference equipment and have been validated by comparisons with certified ARPAE MSR data. The BME280 is a high-linearity and high-accuracy air temperature, humidity, and pressure sensor. Respectively, its pressure operation range is 300 hPa to 1100 hPa, −45 °C to 85 °C for temperature, and 0% to 100% for humidity. It features an extremely fast response time $\tau_{63\%}$ of 1 s, which allows for consistent oversampling compared to the current application time granularity [23].

The laser scattering-based SPS30 PM sensor allows for mass concentration and number concentration sensing. The sensor encapsulates a miniaturized fan and a High-Efficiency Particulate Air (HEPA) filter to reduce optical particle contamination; it also runs its fan at full speed for 10 s every 7 days and at startup as an automatic cleaning procedure. The mass concentration measurement range is from 0 to 1000 $\mu g/m^{3}$. As discussed in [24,25], the SPS30 is an optical particle counter (OPC) optimized for PM2.5 and smaller particle analysis. In fact, Sensirion PM sensors are calibrated and aligned with regularly maintained high-end reference instruments (e.g., the TSI ptical Particle Sizer Model 3330, manufactured in Shoreview, MN, USA, by TSI Incorporated), or the TSI DustTrak™ DRX 8533, manufactured in Shoreview, Minnesota, USA, by TSI Incorporated) only for the 2.5 μm particles size. Furthermore, as reported in the sensor specification statement from the producer, the PM4 and PM10 output is not measured directly, but is estimated from smaller particle counts using typical aerosol profiles. This behavior is also confirmed by the SPS30 detection range being identical for 1 μm and above, thus warning about the use of the sensor for sensing bigger particles [26].

### 2.2. Measurement Site and Reference Instrumentation

**Figure 1:**
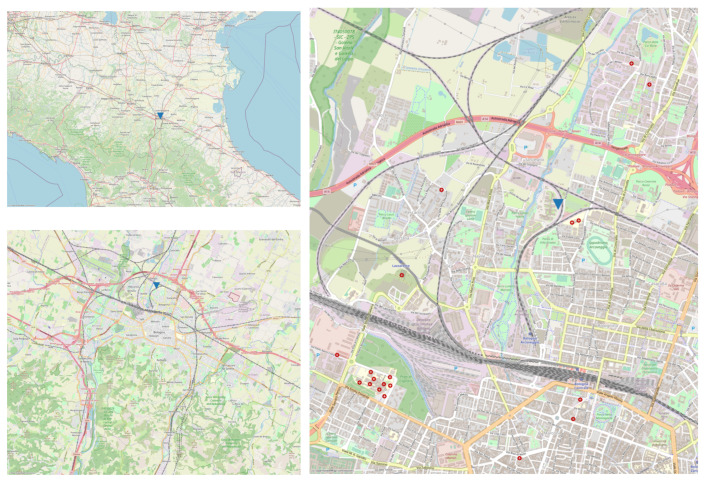
Location of the reference station site in Bologna, in decimal degrees (DD): (44.523698, 11.340034). The figure on the top left shows the position of the site (shown by a blue triangle) in the city of Bologna in northern Italy. The figure on the bottom left shows the position of the site (shown by a blue triangle) in the city of Bologna. The figure on the right shows a zoomed area in the city of Bologna where the site, shown by the same blue triangle, is located.

The reference site is located in northern Italy, inside the research area of the Center for National Research (CNR) in Bologna (see Figure 1). The site is classified as an urban background station. Measurements from this instrumentation will be referenced as the ARPAE System (AS) in the following sections. The reference measurements chosen were performed with an OPC FAI (Multichannel Monitor, FAI Instrument, Rome, Italy) which classifies particles in 8 size intervals from 0.28 μg to 10 μg, is equipped with a 10 μg inlet head, and operates with a flow rate of 1 l/min. The measurement principle is laser scattering: the sensor uses a 35 mW laser diode as the light source and a mirror collecting system elliptical. The light diffused by the particles and collected by the elliptical mirror is concentrated in a photodiode that converts light energy into an electric current. The air sample is transferred to the mixing chamber where it is diluted with clean and dehumidified air (free of particles and with a relatively low humidity level); a smart heater is placed in the diluter along the mixing chamber that is automatically operated only when needed. The OPC Multichannel Monitor is therefore equipped with a temperature and relative humidity sensor in the external environment and is protected from direct solar radiation and from rain, and there is one inside the instrument to detect temperature and humidity relative to the diluted sampling air that passes through the Laser Sensor. Furthermore, the FAI instrument implements a tool (Zero Test) to verify that the sensor provides “zero” counts in the presence of particle-free air. This test also makes it possible to verify that there is no infiltration of external air in the dilution circuit (in the first hour, 15 tests in zero minutes are dropped every day). The OPC FAI also provides measurement of estimated PM (PM1, PM2.5, PM10) and is integrated with a Swam Dual Channel (SWAM 5a DC, FAI, Rome, Italy) that determines the daily mass concentration of PM2.5 and PM10 with a β-ray attenuation method using a low volume (2.3 m^3^/h). The integration gives an automatic correction with real mass values supplied by the SWAM DC with a self-learning procedure. Conversion from number to PM mass concentration is performed using the algorithms provided by the FAI constructors.

### 2.3. Measurement Campaign with Sensors Arrangement

The experimental campaign started on 18 May 2021 at 12.00 and ended on 21 May 2021 at 12.00. The total duration of the test was three full days, providing 72 points for the chosen temporal resolution of 1 h. The latter was defined according to the typical time resolution of the two classes of instruments. Reference instrumentation provides data with a sample rate of 1 min, but verified measures are usually provided as a daily average. On the other hand, low-cost SoS can output data at a sample rate of 10 s. Given the different time granularities of the two systems, we opted for a 1 h time average, which gave a total of 72 samples calculated by averaging the 4320 min samples available (26,000 for the LCSoS). However, a 24 h average of the data measured during parallel measurement with the reference method shall always be calculated and reported [27]. Only two central days of the measurement period are suitable for such a comparison, as given in Table 1, also because a daily average should be considered valid only if 75% of the hours are covered; this is not the case for the first and last days. The proposed study covers a relatively short period (72 h) during which measurements taken by low-cost sensors every 10 s were compared with measurements taken by reference sensors every 60 s. The number of measurements taken, upon which the comparison is based, is thus relatively large (4320 values), but refers to a limited time interval and under weather conditions that do not represent the entire year. During those three days, the weather conditions were homogeneous, according to the report obtained from the ARPAE database [22]. The three days were characterized mainly by sunny or partly cloudy weather with temperatures in the range of 9–23 °C and a wind velocity in the range of 5–17 km/h. For this reason, we believe that the comparison has statistical relevance only for those specific weather conditions, and we can consider the procedure shown as just a single data point in the proposed validation. The experimental setup was built balancing the needs of the experimental campaign with those of the sensors. To ensure the best performance of the SoS, we extended the design and assembly guidelines provided by Sensirion for the SPS30 to the whole system. The most important are as follows:

- Good coupling with ambient air and proper exposition to external conditions;
- Avoidance of exposure to direct sunlight or external heat sources.

**Figure 2:**
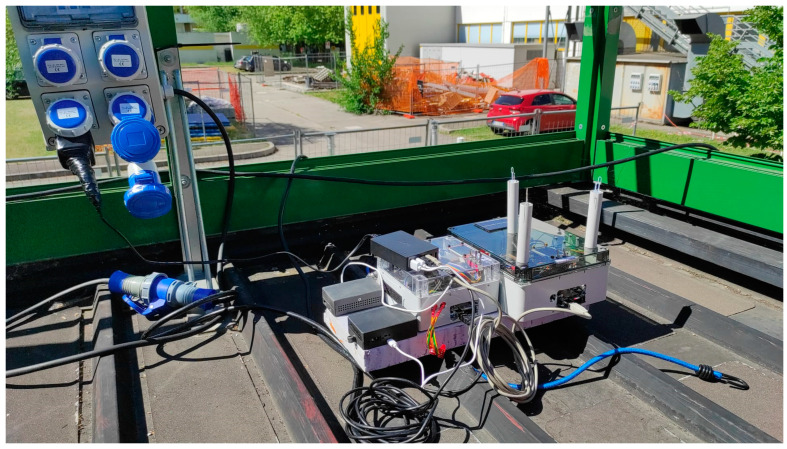
Experimental setup for the LCSoS on the reference station roof.

The low-cost SoS was placed near the sampling inlet of the reference station. A 4 cm thick thermal insulation layer was used at the bottom and top to provide shading and thermal insulation. A pierced plastic shell was used to connect the two planes, providing support for the top plane but allowing good air exchange in the sampling volume, as shown in Figure 2.

These sensors can be used for pollutant concentration measurements in vehicles, as the sensors were embedded in proper containers, as shown by Figure 2. However, here the systems are steady to compare the measurements with those taken by the sensors placed in a station. In order to reduce the disturbances during the measurements in a moving vehicle, the case should be designed in order to avoid a direct impact of the eternal air on the sensors. In our case, the air inlets are on the same side of the case as the back of the vehicle.

### 2.4. Methods Used to Acquire and Manipulate Data

**Figure 3:**
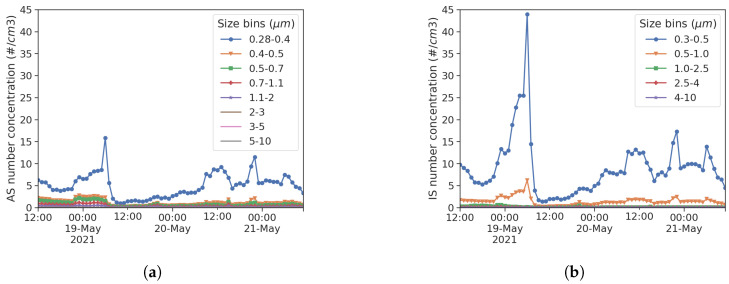
AS row buckets in 8 size channels (**a**) and IS row buckets in 5 size channels (**b**).

The two acquisition systems based on Arduino Mega 2560 were programmed using the standard Arduino IDE. The following specific libraries were used for the sensors mentioned in the previous section: sps30.h, DallasTemperature.h, Adafruit_SGP30.h, Adafruit_BME280.h, DFRobotHCHOSensor.h, and TinyGPS++.h. Each acquired data point was saved on an SD memory card together with a time reference, synchronized in both systems by the reference clock signal received by GPS from both systems. An Exploratory Data Analysis (EDA) approach was applied to the data set, thus providing insight into the definition of the problem and the imposition of the model only after analyzing the data [28]. The open source tools chosen for the EDA were python 3.8 and several scientific computing libraries (pandas, matplotlib, numpy, and scipy above all). The procedures used are more graphical than quantitative, as required by the EDA approach. When comparing multiple particle sensing devices, their built-in size buckets must be considered. Reference instrumentation often comes with a higher size resolution than LCSs, and an accurate grouping of size bins becomes inevitable [29]. Table 2 shows the grouping adopted in this study for EDA and confirmatory data analysis, while Figure 3 shows channel splitting for reference and low-cost instrumentation, respectively.

### 2.5. Time Granularity

Choosing the best time granularity for data analysis is not a simple task. Some authors suggest defining it in the EDA phase and before data analysis is performed; moreover, they warn against the use of statistical significance as a time granularity selection metric, as it can lead to misinterpretation of the results [30,31]. In this study, the following factors are considered: total test duration, different time scales between SoS and official data, and the field of application. A compromise is needed between the best time scale for automotive applications (in minutes or less) and the time scale of validated data from ARPAE (daily values with a six-month validation process). A common approach for ambient validation of PM data is to perform analysis on hourly averaged results [29].

### 2.6. Mass Conversion Correction

**Figure 4:**
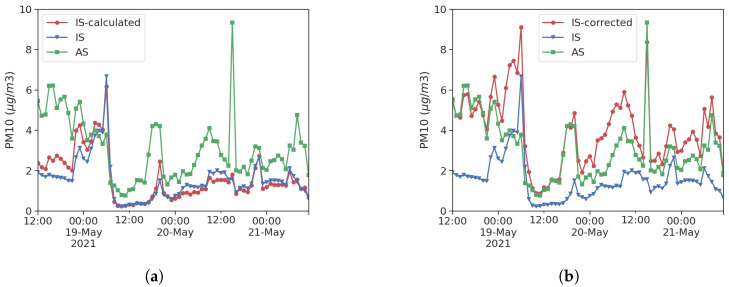
Particle count to mass concentration conversion: (**a**) calculated from particle count according to Equation (1); (**b**) corrected according to reference station average particle mass.

Before delving into the results of mass conversion, it is worth recalling how the mass concentration can be calculated from particle counts, together with associated challenges. Mass conversion adopted in OPC is based on the assumption that particles are spherical and that there is a common density in each size bin; in practice, this is made according to the following equation [13], based on the assumption that particles are spherical and that there is a single density in each size bin:

(1) $$PM_{i}=10^{-9}\;\cdot \;\rho_{p,i}\;\cdot \;\frac{\pi }{6}\;\cdot \;{\tilde{d}}_{i}^{3}\;\cdot \;NC_{i}$$

where *i* is the particle size bin, PM is the mass concentration in μg/m^3^, $\rho_{p,i}$ is the particle average density, $\tilde{d}$ is the median particle diameter in nm, and NC is the number concentration for a given size bin. It is immediately clear from Equation (1) that a good estimation of $\rho_{p,i}$ as the input parameter for the conversion algorithm is crucial. A widely adopted value is 1.65 g/cm^3^ [32], but its variability in time and space must be carefully considered after relocation. The mass conversion based on Equation (1) requires $\tilde{d}$ to be known in each size bin; this information is not always available for LCSs. Sensirion SPS30 provides a typical particle size output $d_{typ}$ that is correlated with the weighted average of the number of concentration bins measured with a reference particle sizer. Substituting $d_{typ}$ in Equation (1) leads to the calculation of a typical particle mass, which can be used to perform a manual conversion whose results are shown in Figure 4. Pearson correlation between LCS and AS increases from 0.43 to 0.60 in this case. An alternative approach proposed in this study instead relies on the calculation of a constant ratio, $\frac{PM_{i}}{NC_{i}}=m_{p,i,AS}$, between mass and particle counts in the chosen bin from reference instrumentation data. The defined quantity can be seen as the average particle mass used to calculate a corrected mass concentration from the number concentration of the LCS. The latter is a direct and more reliable measurement, as shown by Figure 4. With the correction method according to the reference station’s average particle mass, the Pearson correlation of PM values between LCS and AS increases from 0.43 to 0.81, a value that is again comparable to the correlation between particle number concentration.

## 3. Results

**Figure 5:**
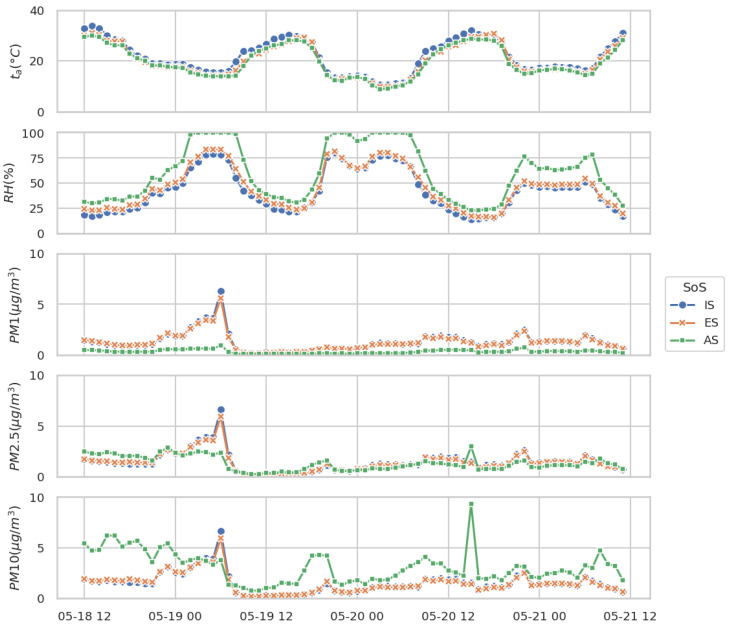
Comparison between hourly data acquired from the two LCSs (IS and ES) and from reference instrumentation (AS). Variables of interest are air temperature, relative humidity, PM1, PM2.5, and PM10.

A qualitative overview of the SoS performance is shown in Figure 5 for the duration of the test.

**Figure 6:**
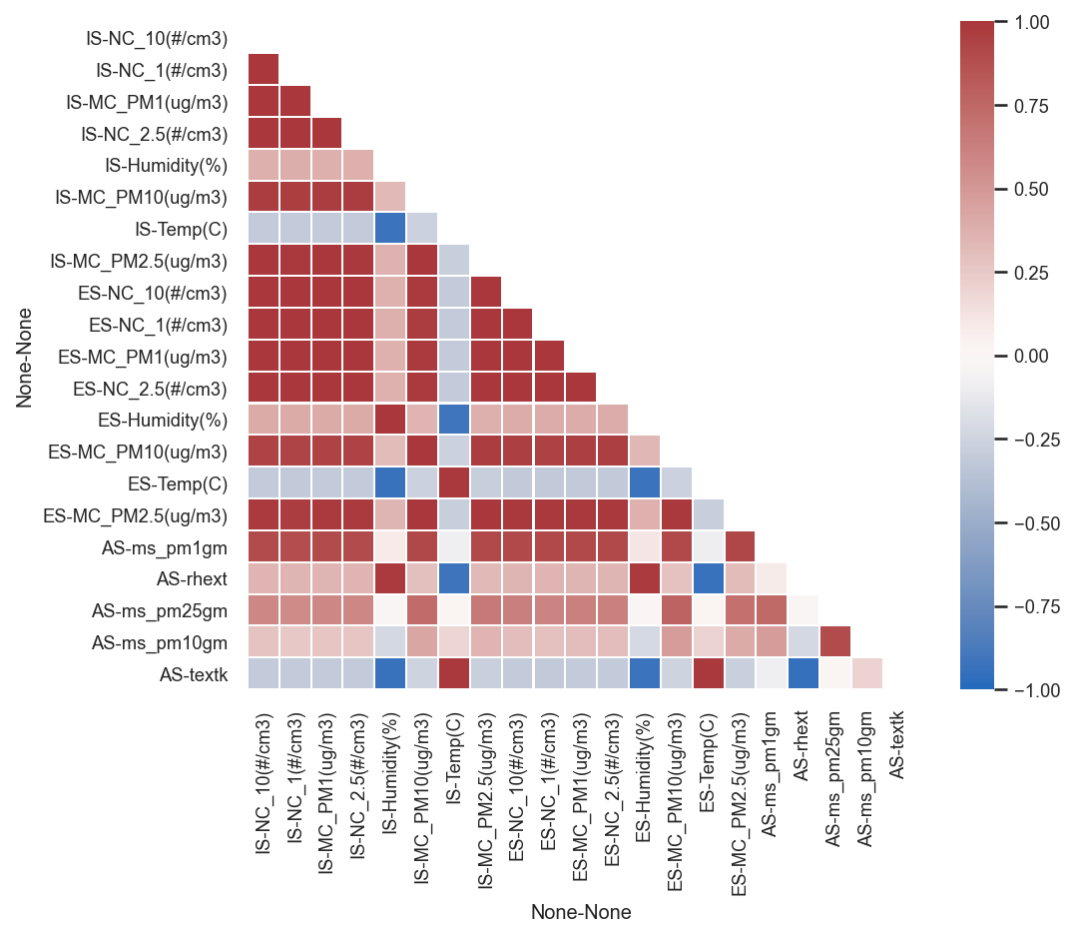
Global correlation heatmap.

The two LCSs show an excellent consistency between themselves, as shown in Figure 5. This result has been shown by other studies [26]. Good agreement on temperature data is also found, while for the other measurement a statistical analysis is needed. The innovative approach adopted in this paper starts from a qualitative analysis based on a correlation heatmap in order to choose the variables. The global correlation heatmap is shown in Figure 6. The figure shows the correlation between multiple variables as a color-coded matrix. Five variables of interest were selected considering their overall correlation with the concentration of mass of particulate matter: air temperature, relative humidity, PM1, PM2.5, and PM10. In contrast, variables that showed a poor Pearson correlation coefficient R were ignored.

**Figure 7:**
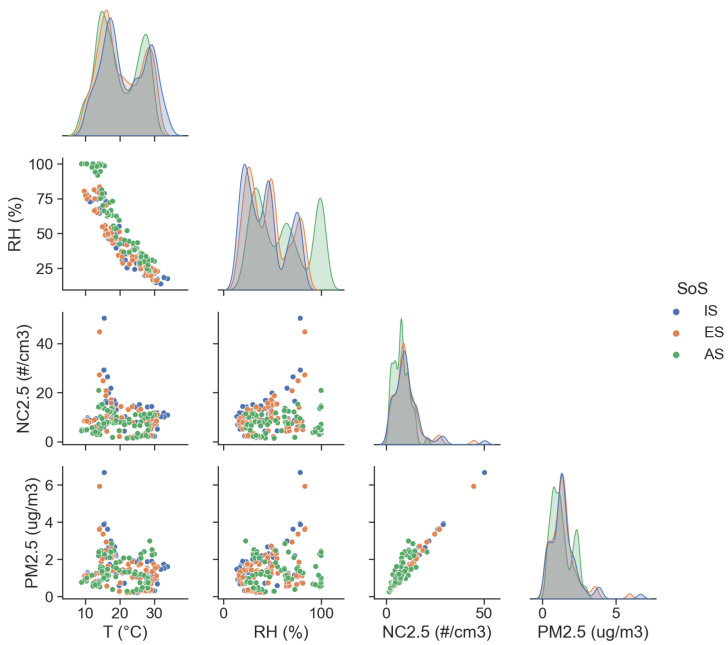
Univariate kernel density estimation plots (diagonal) and bivariate scatter plots (off diagonal) of $t_{a}$, RH, NC2.5, and PM2.5 distributions.

Figure 7 shows univariate kernel density estimation plots (diagonal) and bivariate scatter plots (off diagonal) of $t_{a}$, RH, NC2.5, and PM2.5 distributions.

The plots give an estimate of the probability density functions of the variables based on the measured data points. Figure 7 shows the peaks that correspond to the modes of the distribution. Particular attention must be paid to RH, as it has a direct impact on the sensing mechanism of OPCs, and poor performance at full scale raises an alert. With respect to PM, correlation seems to decrease with the range from smaller particle sizes to larger ones. In the previous sections, a quantitative analysis of SoS performance was given, with a focus on the effects of time granularity, relative humidity, mass conversion from particle counts, and size detection response. Prior to that, an accent must be put on the small PM values observed during the test, thus making the instrumentation operate at the low end of the measurement range.

### RH Sensitivity Analysis

**Figure 8:**
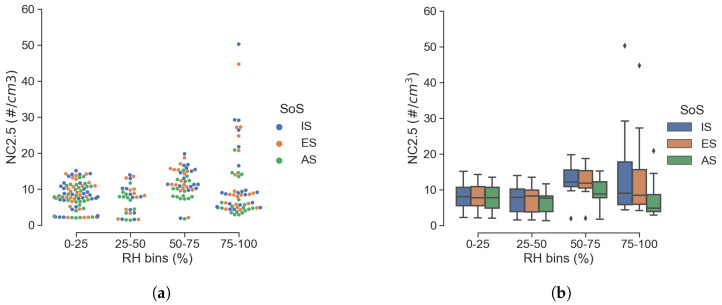
Effect of RH on 2.5μm particle count (**a**) and with data aggregated in 4 RH bins (**b**).

**Figure 9:**
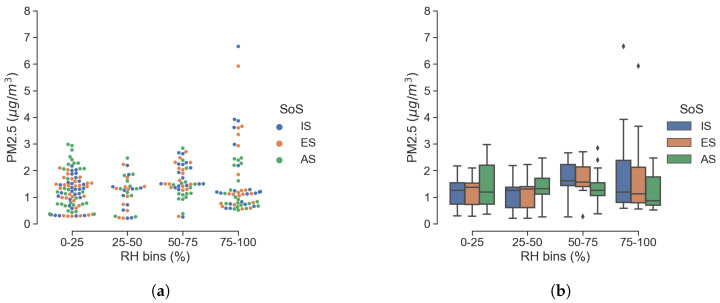
Effect of RH on PM2.5 (**a**) and with data aggregated in 4 RH bins (**b**).

It is well known that among the factors that influence PM measurements, RH plays a major role. This is true to the point that reference gravimetric methods rely on a controlled sampling process in terms of the temperature and relative humidity of the air sample across the filter. These methods are not applicable for LCS and the definition of an RH correction factor is quite common [14]. To evaluate the influence of RH on the sensor response, we can look at the particle sizes in Figure 8 and Figure 9, which show scatter and box plots of number and mass concentration for 2.5 μm. The RH values were aggregated in four uniformly spaced bins, which provided information on the statistical dispersion in each RH bin. Although in agreement among themselves, the low-cost SoS of IS and ES has a wider interquartile range, together with a greater number of outliers; the worst scenario corresponds to RH values greater than 75%.

**Figure 10:**
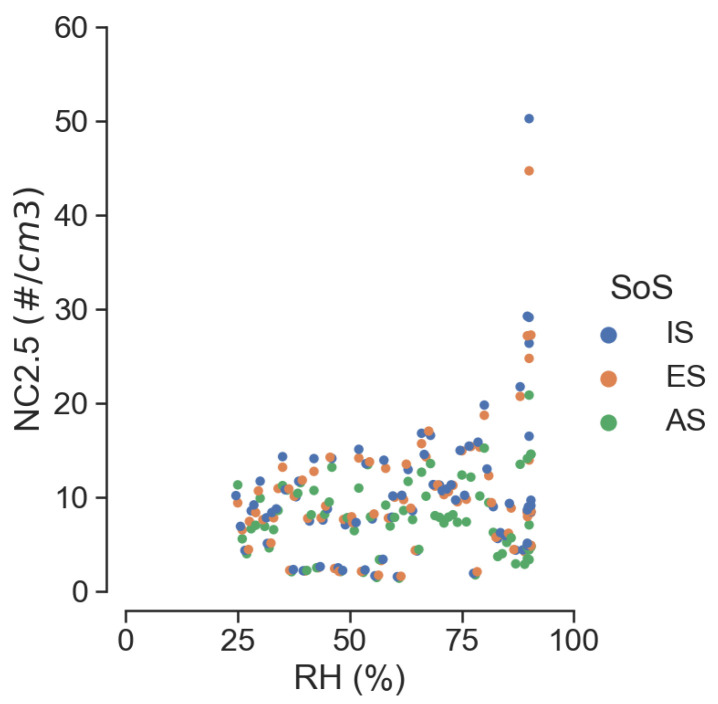
Scatter plot of 2.5 μm particle count values without RH binning.

Similar consideration can be drawn from Figure 10, which reports the same quantities but without binning. An increased dispersion of the data is noticeable with growing RH values; also, in this case, higher particle counts associated with high RH values do not fit well with the reference system AS. Note that the dispersion of data points increases in the transition from particle count to particle mass, probably as a result of the theoretical mass conversion adopted in OPCs.

**Figure 11:**
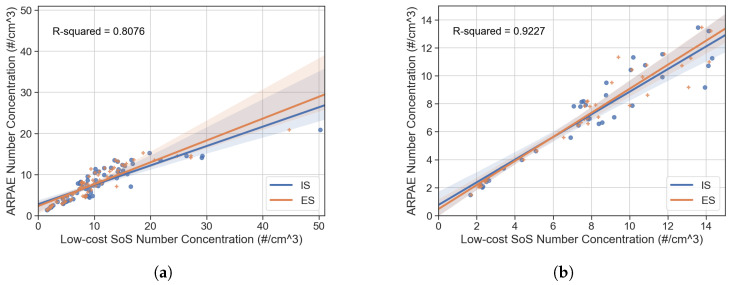
Linear regression for 2.5 μm particle size using the full data set in terms of RH 0–100% (**a**) or a reduced data set with RH 0–50% (fewer data points) (**b**).

In order to quantify the impact of the predictor variable RH on the response variable NC2.5, a univariate linear regression was adopted. The results of the ordinary least squares (OLS) analysis are reported in Figure 11, again showing that sensor performance degrades at high RH values. The improvement in $R^{2}$ approaches 0.11 when the values of RH above 50% are dropped from the data set.

## 4. Discussion

In this study, an innovative calibration procedure is shown to qualify low-cost sensors capable of a high spatiotemporal resolution through a field comparison against the reference instrumentation operated by ARPAE. The main factors influencing the output of the particle sensors were first measured and then identified with an Exploratory Data Analysis (EDA) approach. Their impact in terms of data quality and correlation with reference instrumentation was also analyzed. The LCSoS is made up of an open-source Arduino platform and a commercially available LCS. Open-source software tools were used for acquisition and post-processing algorithms, which further supported the repeatability of the experiment and spreadability of the device. The results show that the system has relatively good performance with respect to air temperature, relative humidity, and particle number concentration ($d_{p}\leq 2.5\;\mu m$) with a determination coefficient approaching $R^{2}=0.92$ for the reduced data set, as also confirmed by other studies [29]. Relative humidity has a strong impact on performance indicators and correction techniques are strongly recommended [16,17]. If few works dealt with Sensirion SPS30, even fewer dealt with mass conversion [13,33]. We compared three conversion approaches: the sensor built-in output, a mass concentration calculated from particle concentration, and a mass concentration corrected according to reference instrumentation data. Both the conversion techniques provide an improvement in the overall range of Pearson *R* from 0.2 to 0.4 on the PM10 value. It is also important to note that the correlation of PM with daily values from the verified ARPAE network is quite poor; on the other hand, good quality and consistency of the data among LCSoS were observed. An IAQ monitoring system such as the one proposed in this research can be subject to frequent relocation (moving measurement); this can lead to calibration issues that must be addressed. More sophisticated calibration models are supposed to provide more effective calibration procedures, but require longer training [9,14] and might result in unpractical methods for automotive and high-spatiotemporal-resolution applications. For this reason, we opted for a short three-day measurement period and well-established, yet straightforward, performance assessment procedures. Further research on these aspects is essential, as low-cost sensors (LCSs) can provide valuable insights. However, their reliability is still not on par with reference instrumentation. The aim of this work is to see a broader adoption of validated low-cost sensors in the automotive sector. This expansion has the potential to greatly improve the monitoring of pollution in urban areas.

## Figures and Tables

**Table 1:** Measured quantities and daily summary. IS and ES are the low-cost sensors. AS is the ARPAE reference system and MSR is the main reference site.

Variable	Specifications		Summary (Day 2, Day 3)			
(Unit)	Description	Sensor	IS	ES	AS	MSR
$t_{a}\;{(}^{\circ }C)$	Air temp.	BME280	(21.0, 20.8)	(19.5, 19.6)	(19.1, 18.9)	(18.7, 15.8)
RH(%)	Air rel. hum.	BME280	(51, 43)	(55, 46)	(71, 62)	(42, 57)
$PM_{1}\;\left(\mu g/m^{3}\right)$	PM1 conc.	SPS30	(1, 1)	(1, 1)	(0, 0)	n.d.
$PM_{2.5}\;\left(\mu g/m^{3}\right)$	PM2.5 conc.	SPS30	(1.5, 1.4)	(1.4, 1.3)	(1.1, 1.1)	(5.2, 2.4)
$PM_{10}\;\left(\mu g/m^{3}\right)$	PM10 conc.	SPS30	(2, 1)	(1, 1)	(2, 3)	(15, 6)
$NC1\;(\#/{\mathrm{cm}}^{3})$	Number conc.	SPS30	(11, 11)	(10, 10)	(6, 8)	n.d.
$NC2.5\;(\#/{\mathrm{cm}}^{3})$	Number conc.	SPS30	(11, 11)	(10, 10)	(6, 8)	n.d.
$NC10\;(\#/{\mathrm{cm}}^{3})$	Number conc.	SPS30	(11, 11)	(10, 10)	(6, 8)	n.d.

**Table 2:** Particle count bucket grouping.

AS		IS, ES	
Channel	Size Range (μm)	Channel	Size Range (μm)
1	0.28–0.4	1	0.3–0.5
2	0.4–0.5		
3	0.5–0.7	2	0.5–1.0
4	0.7–1.1		
5	1.1–2.0	3	1.0–2.5
6	2.0–3.0		
7	3.0–5.0	4	2.5–4
8	5.0–10	5	4.0–10

## Data Availability

Data are contained within the article.

## Abbreviations

ARPAE	Agenzia regionale per la prevenzione, l’ambiente e l’energia dell’Emilia-Romagna
AS	ARPAE System of Sensors
BEV	Battery Electric Vehicle
CNR	Center for National Research
DD	Decimal Degrees
EDA	Exploratory Data Analysis
ES	Low-Cost Measurement System 1
IAQ	Internal Air Quality
IS	Low-Cost Measurement System 2
LCS	Low-Cost Sensor
LCSoS	Low-Cost System of Sensors
ML	Machine Learning
MRS	Main Reference Site
NC	Number Concentration
OLS	Ordinary Least Squares
OPC	Optical Particle Counter
PM	Particulate Matter
PM1	Particulate Matter with a Diameter of 1 μm (µm) or less
PM2.5	Particulate Matter with a Diameter of 2.5 μm (µm) or Less
PM10	Particulate Matter with a Diameter of 10 μm (µm) or Less
RH	Relative Humidity
SoS	System of Sensors
$t_{a}$	Ambient Temperature (°C)
